# Integrating multiomics data accelerates elucidation of plant primary and secondary metabolic pathways

**DOI:** 10.1007/s42994-022-00091-4

**Published:** 2023-01-11

**Authors:** Feng Zhu, Weiwei Wen, Yunjiang Cheng, Saleh Alseekh, Alisdair R. Fernie

**Affiliations:** 1 National R&D Center for Citrus Preservation, Hubei Hongshan Laboratory, National Key Laboratory for Germplasm Innovation and Utilization for Fruit and Vegetable Horticultural Crops, Key Laboratory of Horticultural Plant Biology, Ministry of Education, Huazhong Agricultural University, Wuhan, 430070 China; 2 Max Planck Institute of Molecular Plant Physiology, Am Mühlenberg 1, Potsdam-Golm, 14476 Germany; 3 Center of Plant Systems Biology and Biotechnology, Plovdiv, 4000 Bulgaria

**Keywords:** Metabolome, Transcriptome, Genome, Crop improvement

## Abstract

Plants are the most important sources of food for humans, as well as supplying many ingredients that are of great importance for human health. Developing an understanding of the functional components of plant metabolism has attracted considerable attention. The rapid development of liquid chromatography and gas chromatography, coupled with mass spectrometry, has allowed the detection and characterization of many thousands of metabolites of plant origin. Nowadays, elucidating the detailed biosynthesis and degradation pathways of these metabolites represents a major bottleneck in our understanding. Recently, the decreased cost of genome and transcriptome sequencing rendered it possible to identify the genes involving in metabolic pathways. Here, we review the recent research which integrates metabolomic with different omics methods, to comprehensively identify structural and regulatory genes of the primary and secondary metabolic pathways. Finally, we discuss other novel methods that can accelerate the process of identification of metabolic pathways and, ultimately, identify metabolite function(s).

## Introduction

Metabolites are fundamental components of the plant cell. These compounds play vital roles not only in plants as energy suppliers, signaling regulators, and enzyme cofactors, but also for human health, given that they supply carbohydrates, fatty acids, proteins, vitamins and minerals, alongside bioactive secondary metabolites (Canarini et al. 2019; Zaynab et al. 2019). Based on former estimates, the plant kingdom produces some 1 million metabolites for different functions (Alseekh and Fernie 2018). Moreover, these metabolites are the most important bio-markers, reflecting the exact physiological status of the plant (Afendi et al. 2013; Fang et al. 2019). Among these metabolites, some of them, such as sugars, organic acids, and amino acids directly participate in plant basic metabolism and maintain fundamental biological processes, including functional macromolecule biosynthesis. Such metabolites are regarded as primary metabolites. Moreover, other metabolites, such as polyphenols, terpenoids, and alkaloids, function as important regulators, not only of different plant growth and development processes but also against the biotic and abiotic stresses and further exhibit high bioactivities, thereby allowing them to augment defense to human inflammation, cardiovascular diseases, and cancer. Such plant metabolites are named as secondary metabolites or specialized metabolites (Tiwari and Rana 2015; Tohge et al. 2014; Verpoorte and Memelink 2002).

Considering the diversity and importance of metabolites, an important issue in metabolomic research is the development of a high-throughput and exact identification and qualification method. Recently, owing to the rapid innovation of liquid/gas chromatography and high-sensitive mass spectrometry, global analyses, conducted across different plant species, have identified many thousands of metabolites (Obata et al. 2020; Peng et al. 2017). To advance knowledge concerning metabolites, improving our understanding of metabolic pathways of biosynthesis and catabolism has recently attracted considerable attention. Indeed, many advances have been achieved by incorporating large-scale genome and transcriptome analyses (Li et al. 2020; Zhu et al. 2022a).

In this review, we summarize the importance of metabolites and the profiling methods to study the different kinds of metabolites. We also stress the importance of research that integrates the combination of metabolomic, genomic and transcriptomic datasets. We then discuss other efficient strategies to better decode the primary and secondary pathways of various crops.

## Importance of the plant metabolome and research strategies employed for its evaluation

As mentioned above, metabolites can be divided into two major classes, primary metabolites and secondary metabolites (Wang et al. 2022a); however, the distinction between these is, at times, blurred (Erb and Kliebenstein 2020; Fàbregas and Fernie 2021). Both classes play important roles in plant development, response to stress, and reproduction. However, the profiling methods used to obtain information on their levels are slightly different.

### Primary metabolites

As the most well-studied primary metabolites, organic acids and sugars are the key components of the tricarboxylic acid cycle (TCA cycle) and glycolysis pathways (Krebs 1970). These pathways not only provide the main energy source, and some components used for the generation of signaling molecules, but also produce different substrates for the synthesis of other important primary metabolites, such as amino acids and fatty acids (Sweetlove et al. 2010; Zhao et al. 2021).

As an example, exogenously applied sugars can increase the fresh weight, vitamin C, soluble protein, and sugar contents of pea sprouts (Tan et al. 2022). The availability of cellular sugars reflects the plant’s carbon nutrient status, which then induces the functions of nutrition sensing, through such pathways as the hexokinase glucose sensor, the trehalose 6-phosphate signal, and the Target of Rapamycin kinase pathway, to regulate plant growth and development processes (Smeekens et al. 2010).

In addition, 2-oxo-glutarate is an important intermediate in the TCA cycle but can also be converted to glutamate that can then be transferred to other amino acids (García-Gutiérrez et al. 2018; Liepman and Olsen 2003). Moreover, isotope labeling experiments have demonstrated that pyruvate, the key component of glycolysis, is the immediate precursor for the synthesis of alanine (Kennedy and Laetsch 1974). The accumulation of some primary metabolites, such as proline, can significantly enhance plant tolerance against the abiotic stresses resulting from the global climatic change (Ghosh et al. 2022). Besides sugars, organic acids and amino acids, fatty acids, and their derived lipids, are predominant components of both the plasma membrane and photosynthetic membranes (Li et al. 2016). Similarly, fatty acid metabolism also affects both cuticular wax biosynthesis and pollen fertility, which are important processes for plant adaption to a terrestrial environment and propagation (Millar et al. 1999; Wang et al. 2022b; Zhang et al. 2021a).

### Secondary metabolites

Based on their molecular structures and biological functions, secondary metabolites can be divided into various classes, such as terpenoids, polyphenols, alkaloids, non-ribosomal polypeptides, and enzyme cofactors (Tohge et al. 2014; Verpoorte and Memelink 2002). Given that their sessile character has considerably limited the communication of plants with other plants and animals, some metabolites of the terpenoids, such as terpenes, are volatile and play important roles in the communication between plants with pollinators, seed dispersers, and signal transduction between different plants (Dudareva et al. 2013). Another example is the representative C40 terpenoids; these carotenoids play vital roles in plant photosynthesis, photoprotection, and development, and are important in the synthesis of pigments associated with fruit appearance quality (Nisar et al. 2015).

**Fig. 1:**
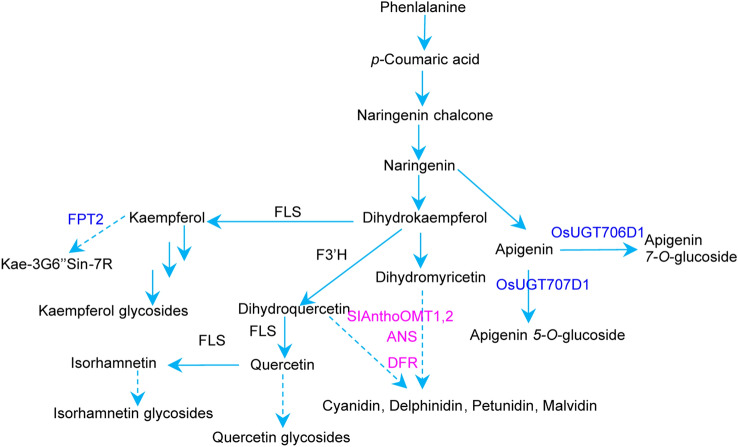
Representative decoded flavonoids-related genes addressed in the present review. Purple and blue-marked genes were identified in Zhang et al. (2020), Peng et al. (2017) and Tohge et al. (2016). Kae-3G6″Sin-7R: flavonol-3-*O*-(2″-*O*-rhamnosyl-6″-*O*-sinapoyl) glucoside-7-*O*-rhamnoside

Polyphenols are the most studied secondary metabolites, and are derived from phenylalanine, via the shikimate/phenylpropanoid pathway. After condensation with malonyl-CoA, and modifications including methylation and glucuronidation, phenylalanine can be transferred to representative polyphenols. such as quercetin 3-*O*-rutinoside (rutin) and anthocyanin. Among the polyphenols, flavonoids are the most studied class and generally contain two aromatic rings. The different modifications, on these aromatic rings, led to the super diversity of the flavonoids, which have been estimated to contain over 6000 compounds (Górniak et al. 2019). Recently, scientists reported that, in *Arabidopsis* flowers, a high accumulation of a class of phenylacylated-flavonols (saiginols) was attributed to protecting the Brassicaceae flower from damage caused by the UV light in sunshine (Tohge et al. 2016) (Fig. 1). As important cereals of the human diet, the three grass crops, maize, rice, and wheat, accumulate some special flavonoids, such as glycosylated flavones. These metabolites also function as UV protectors in these crops (Peng et al. 2017) (Fig. 1).

Besides their importance in food, flavonoids also present in many human beverages, such as tea, and play important roles for human health. For example, as a main bioactive ingredient in green tea, epigallocatechin-3-gallate (EGCG), can repress the infection of influenza virus and other representative viruses, such as HCV and HIV-1, and also affect human-pathogenic yeast strains by affecting the folic acid metabolism of bacteria and fungi (Steinmann et al. 2013). In addition, it is well known that the representative flavonoids, the anthocyanins, play vital roles in human protection against a broad range of diseases (Ciumărnean et al. 2020). To develop tomato fruit with enhanced anthocyanin levels, scientists generated transgenic plants expressing two transcription factors from snapdragon (*Del* and *Ros1*), thereby enhancing, threefold, the hydrophilic antioxidant capacity in the fruit; feeding these transgenic tomato fruit to cancer-susceptible mice significantly extend their life span (Butelli et al. 2008).

**Fig. 2:**
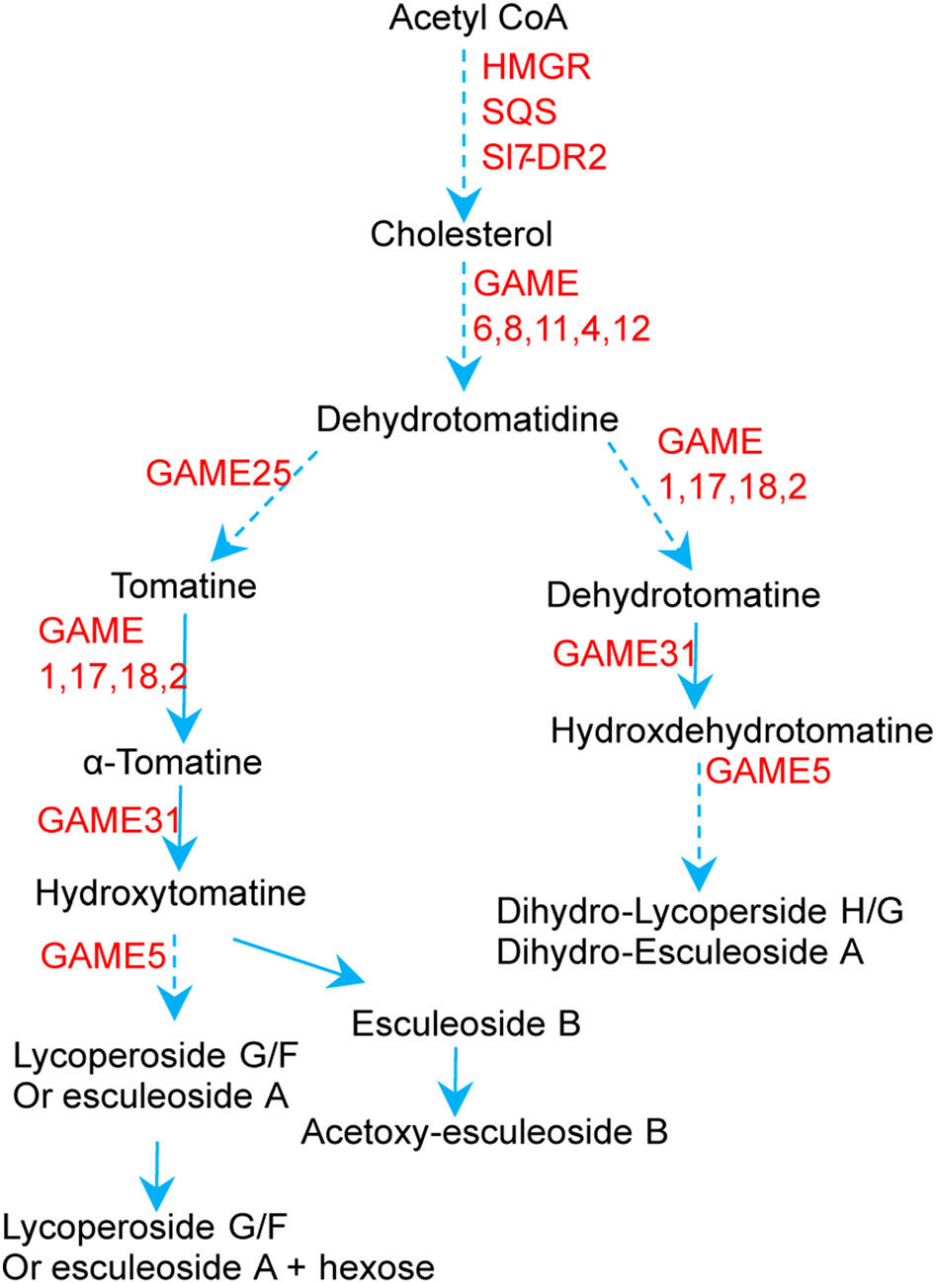
Representative decoded steroidal glycoalkaloids-related genes addressed in the present review. Red genes were identified in Itkin et al. (2013), Itkin et al. (2011), Szymański et al. (2020), and Li et al. (2022). GAME: Glycoalkaloid Metabolism

Alkaloids contain at least one nitrogen atom in a heterocyclic ring and exhibit alkali-like properties. Steroidal glycoalkaloids (SGAs) are special alkaloids accumulating in different plant organs, such as leaves, roots, flowers, fruit and tubers of the Solanaceae family (Alseekh et al. 2020). For example, α-tomatine is the predominantly accumulated SGA in immature tomato fruit and is toxic for insects, fungi, and humans, thereby making it an efficient protection mechanism to reduce unripen fruit loss. Subsequently, during ripening, α-tomatine can be detoxified, via a series of hydroxylation and modification reactions, to produce the human health-promoting chemicals, the esculeosides (Alseekh et al. 2015; Fujiwara et al. 2007; Itkin et al. 2011, 2013; Szymanski et al. 2020) (Fig. 2).

### Metabolite evaluation methods

Given the importance of different metabolites, the research community has played great attention to the methods used in their characterization; here, gas chromatography–mass spectrometry (GC–MS) and liquid chromatograph-mass spectrometry (LC–MS) are the widely used methods. As some primary metabolites are small molecular weight components, which can be easily volatilized after derivatization, gas chromatography-mass spectrometry (GC–MS) is widely used to analyze the primary metabolite contents (Obata and Fernie 2012). The extracted and dried metabolite mixture is first derivatized, by methoxyamine-hydrochloride/pyridine and *N*-methyl-*N*-(trimethylsilyl) trifluoracet-amide, and then analyzed by GC–MS (Salem et al. 2016). To determine the exact metabolite of each fraction, the small fragments are detected by time-of-flight (TOF)-MS, which can quickly and precisely scan the fragment *m*/*z* information. With the help of the established metabolite datasets, such as the Golm metabolic databases (GMD) (Kind et al. 2009; Kopka et al. 2005), efficient annotation and analysis of the relative metabolite content can be analyzed, based on the retention time, mass spectra and peak area information.

The reverse phase column exhibits the highly efficient separation capacity for similar-structured and a broad range of metabolites. Their coupling to LC–MS systems has been employed to identify thousands of fatty acids-derived lipids, carotenoids, polyphenols and alkaloid metabolites (Rupasinghe and Roessner 2018; Salem et al. 2016). The dry-extracted mixture is resuspended, using a suitable solution, such as butanol–methanol mixture or 50% methanol. The resuspended solutions are then directly loaded onto the LC–MS for separation in a liquid phase and electrospray ionization (ESI) prior to detection by a high-sensitive MS, such as an Exactive high-resolution Orbitrap-type MS.

Owing to the variation of LC pump pressure and mass spectra characteristics, it is impractical to build a universal library containing the retention time and mass spectra information for metabolite annotation derived from different labs. In general, based on machine characteristics, an individual-specific LC–MS reference library is established by a lab, through integrating the retention times and mass spectra information for the standard components, useful mass spectra datasets (such as Lipid Maps, http://www.lipidmaps.org/index.html and ReSpect database, http://spectra.psc.riken.jp/menta.cgi/respect/search/fragment) and information in the public domain (Luzarowska et al. 2020; Wang et al. 2019).

In summary, the highly sensitive and accurate metabolite identification method, based on the GC/LC–MS, allows construction of a solid foundation for the study of primary and secondary metabolism, whilst a comprehensive definition of the key genes and regulators of these pathways generally needs more information from both genomic and transcriptomic analyses.

## Integration of genome and metabolome to decode genes involved in the primary and secondary metabolism

The one gene–one enzyme hypothesis of George Wells Beadle indicated that the individual gene-encoded enzymes play important roles in the biosynthesis and catabolism of metabolites (Horowitz 2014), whilst not entirely applicable in plants, due to the commonality of multiple isoforms of the same enzyme, there remains some value in the combined comprehensive analysis of the association of genome and metabolome variations, which we anticipate will still accelerate elucidation of the metabolic pathways. During evolution and the domestication of crops, variations in genes that encode for enzymes may change their amino acid sequence and directly affect enzyme activity. Therefore, to decode the important genes involved in primary and secondary metabolism, scientists traditionally carried out cDNA cloning using secondary metabolite-rich tissue or tissues collected from crosses derived from two parents with extreme phenotypes, and then conducted map-based cloning analysis of the biparental populations (Shi et al. 2020; Wang et al. 2008). Based on the analysis of amino acid content and quantitative trait locus (QTL) mapping of 190 recombinant inbred lines (RILs), some 80 individual QTL were identified for content of 19 amino acids, with a relatively strong QTL cluster, comprised of 19 individual QTL, being detected on chromosome 1 (Wang et al. 2008).

Although many QTL have been identified using RILs, such a population, which segregates concurrently for many QTL dispersed throughout the genome, may cause huge variances in the following statistical analysis and, thereby, reduce the effects of one another, to limit the genetic resolution of these quantitative traits (Zamir 2001). To resolve this limitation, introgression lines (ILs), which cover the entire genome and each line segregates for a single region, have been used for QTL mapping. In such analyses, the phenotype variations are associated with the introduced-targeted segment, which assists in focusing on the analysis of QTL and genes located in the introduced-targeted segment, and this can significantly improve the efficiency of QTL identification (Eshed and Zamir 1995; Schauer et al. 2006).

As an example, total soluble content of tomato fruit is a complex phenotype and the analysis, among a population of 76 segmental ILs of wild species *Solanum pennellii* into the cultivated tomato (*S. lycopersicum* M82), identified a flower- and fruit-specific invertase (*LIN5*), located in the QTL*-Brix9-2-5* (Fridman et al. 2004). In addition, the same population of ILs was used to identify 338 putative mQTL for flavonoids and steroidal glycoalkaloids in the tomato seed (Alseekh et al. 2020).

The above-mentioned populations were based on the genetic resources from two genotypes; however, during the evolution and domestication of crops, hundreds, or even thousands of genotypes of each crop have been created, which contain more abundant genetic and metabolic variations for identification of metabolism-related genes. In addition, the development of next generation sequence methods reduced the cost and made it practical to carry out genome sequencing of hundreds, or even thousands, of genotypes. Therefore, recently, many association studies have been performed to explore metabolic and natural population genetic variations (Chen et al. 2014). As there are millions of variations, within the genome of a population, this complicates the identification of the exact association between a specific metabolite and a specific genomic region. To overcome this problem, a genome-wide association study (GWAS) can be used to explore the connection between a plant metabolite and genetic variation (Mountjoy et al. 2021; Ozaki et al. 2002).

Rice, as one of the most important crops for human food, supplies not only carbohydrates but also secondary metabolites. Using a metabolic genome-wide association study (mGWAS), based on 840 metabolites and some 6.4 million single-nucleotide polymorphisms (SNPs) for a 529 diverse rice accessions panel, identified 2947 lead SNPs that were associated with 598 metabolites, with five candidate genes being further validated with functions in secondary metabolite pathways (Chen et al. 2014).

In addition to SNPs and small indels, structural variations (SVs), such as a big insertion, deletion, copy number variation and chromosomal change, also play important roles in plant metabolism (Hollox et al. 2022; Voichek and Weigel 2020). However, the short sequence length of the first- and second-generation sequencing method significantly limited the identification of the SVs in a population genome. In recent years, the development of a third-generation sequencing method has significantly increased the sequencing read lengths to over 10,000 bp, and has become the ideal method for genome SV detection (Lee et al. 2016). Based on this method, Alonge et al. (2020) identified more than 200,000 SVs for 100 diverse wild and domesticated tomato accessions and identified several associations between SVs with important fruit quality, such as fruit smoky volatile content and fruit size.

## Integration of transcriptome and metabolome to decode genes involved in primary and secondary metabolism

Earlier studies indicated that genomic variation located in a coding region could change the encoded protein amino acid sequence and, thereby, affect protein function. Moreover, other variations located within the promoter or an intergenic region can also significantly affect gene expression and by this manner lead to variation in metabolite abundance across a population (Ye et al. 2017; Zhu et al. 2022a). With the development of low-cost second-generation sequencing methods, a vast torrent of transcriptome and metabolome data have been analyzed to decode the key genes involved in primary and secondary metabolism (Karlova et al. 2011; Zhu et al. 2017, 2020).

As the biosynthesis and catabolism of metabolites results from the ordered sequence of a series of enzymes and their associated regulators, the expression of these genes may exhibit a similar trend with each other and also with the metabolite content (Omranian et al. 2015). Therefore, a comprehensively integrated analysis of metabolome and transcriptome can be carried out to detect the high correlation between an unknown gene expression with the levels of metabolites, and the tightly co-expressed unknown genes with the known function enzyme genes. These correlation analyses can indicate that an unknown gene may be involved in the interesting metabolite pathway, following a method known as the “guilt-by-association” approach (Li et al. 2020; Yonekura-Sakakibara et al. 2008). One example of this approach was the evaluation of the flavonol pathway of wild and cultivated tomato (Tohge et al. 2020). This study increased the number of metabolites recognized in this pathway in tomato from 22 to 44. Similarly, the spatio-temporal metabolome and transcriptome data of 20 major tomato tissues and growth stages were recently integrated to build the MicroTom Metabolic Network and identified several novel transcription factors, such as *SlERF.G3-like*, *SlbHLH114*, regulating the biosynthesis of flavonoids and steroidal glycoalkaloids (Li et al. 2020).

Population scale transcriptome data can provide insight into the expression of genes in different genotypes, and can be integrated with genome variations to reflect the effect of genetic variance on a gene expression phenotype; this is the so-called eQTL (Zhu et al. 2018, 2022b). Recently, Wen et al. (2018) analyzed mGWAS and eQTL in four independent tissues derived from different maize accessions. Based on these large-scale data, 36 loci were identified, and four genes were validated to be involved in trehalose, aspartate, and aromatic amino acid pathways. In addition, the mGWAS method was employed to identify *CsANR*, *CsF3′5’H* and *CsMYB5* as important tea genes involved in biosynthesis of catechins (Zhang et al. 2020) (Fig. 1).

## New methods to decode genes involved in primary and secondary metabolism

Recently, with transcriptome and metabolome technology development, single cell and spatial transcriptomics methods have been established with the potential to provide new insights into metabolism, at a more precise level, for both crop and non-crop plant species (Xia et al. 2022; Zhang et al. 2021b, c). Under the regulation of transcriptional, post-transcriptional, or feedback regulation, metabolite accumulation also exhibits different patterns within various cell types within the same tissue. With the developments and advances in MS, optical spectroscopy, and the fluorescence biosensors, it is slowly becoming practical to simultaneously measure hundreds of metabolites in a single cell (Zenobi 2013).

The mass spectrometry imaging methods, such as secondary ion mass spectrometry and matrix-assisted laser desorption/ionization, are widely used technologies to obtain spatially resolved metabolome information for samples (de Souza et al. 2020; Seydel 2021). Recently, in human cells, based on the gas cluster ion beam secondary ion mass spectrometry (GCIB-SIMS) method, Pareek et al. (2020) successfully visualized the in situ three-dimensional sub-micrometer chemical imaging of de novo purine biosynthesis and identified the enzyme interaction structure, of the purinosome, which can channel the pathway and, thereby, increase the pathway flux yielding purine biosynthetic efficiency. Moreover, ginsenosides are the main bioactive metabolites of popular traditional Chinese medicines, Panax ginseng. Based on matrix-assisted laser desorption/ionization time-of-flight mass spectrometry imaging (MALDI-TOF-MSI), Bai et al. (2016) used a novel approach to identify five different localization types of ginsenosides. Given that different ginsenosides have varied pharmacological effects and can reflect the ages of the ginseng root, this method can provides important cues for the component-specific extraction and the illustration of bioactive metabolites biosynthesis regulation of pharmacological research of herbs.

## Conclusions

**Fig. 3:**
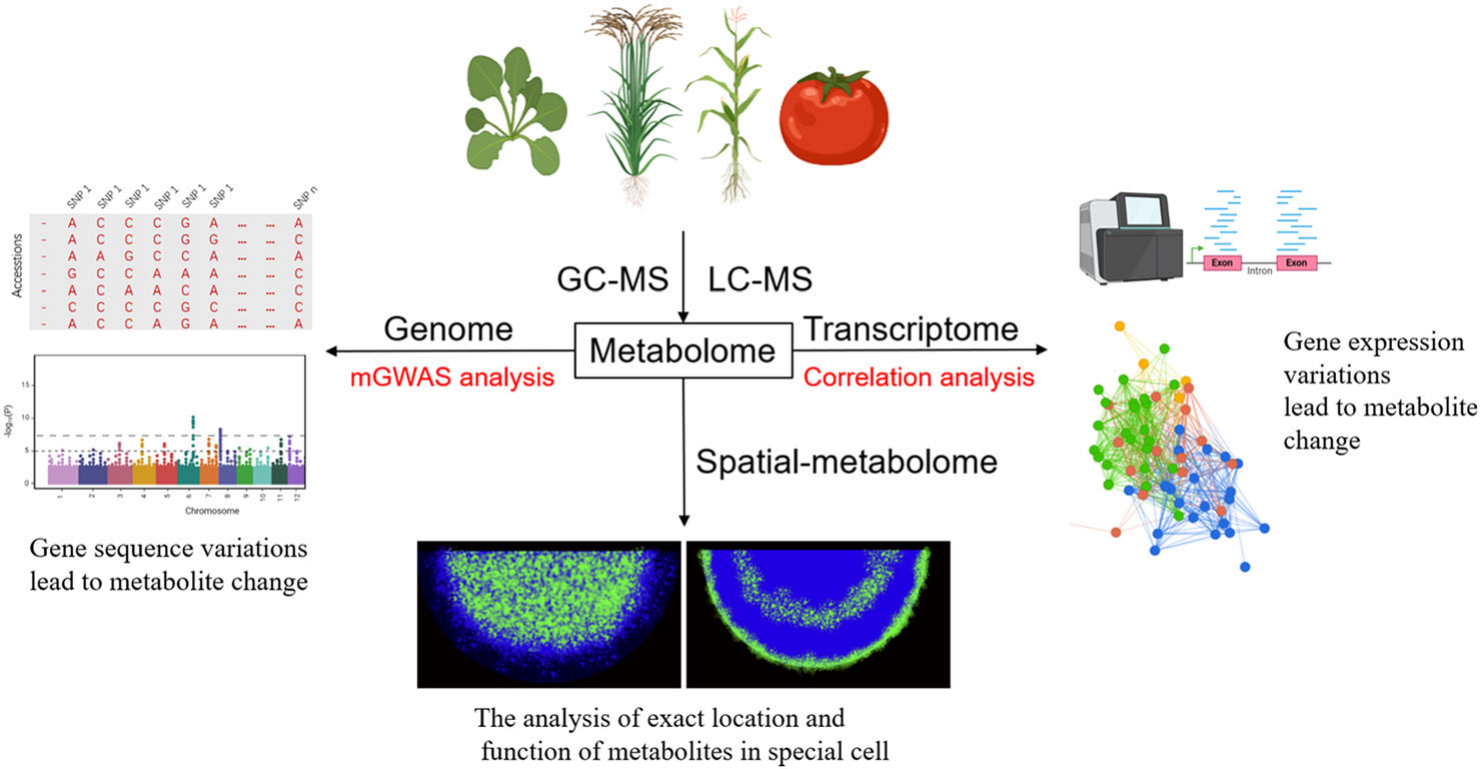
Pipeline of the integrated analysis of metabolome/genome/ transcriptome to identify the primary and secondary pathways and genes. The plant figure was created using BioRender (https://biorender.com/)

In this review, we have summarized the importance of metabolite functions and profiling methods for metabolites alongside the combined analysis of genome and metabolome, or transcriptome and metabolome, or the combination of all three omics, to decode the key genes and regulators of plant primary and secondary pathways (Fig. 3). Although these studies have demonstrated the power of the integrated omics analysis, these strategies are based on accessibility to a large number of natural populations and the inherent genetic variation. They can, therefore, not be applied to some non-crop plant species, such as certain medicinal plants, or even crop species such as banana for which the level of genetic variance is not available in natural populations to facilitate mGWAS analysis. Moreover, using omics methods remains technically difficult for illustrating the regulatory mechanism of metabolite synthesis within special cell types. However, this limitation is beginning to be addressed by the integration of newly developed technologies, such as spatial- transcriptomics and metabolomics and, given the large interest in this research frontier, it seems appropriate to anticipate that this limitation will soon be addressed. Irrespective of these restrictions, the integration of different multiomics data can remarkably accelerate the process towards a complete understanding of the pathway structure and regulation of primary and secondary metabolism in various species.

## Data Availability

Data sharing not applicable to this article as no datasets were generated or analyzed during the current study.
